# *N*-(2-Aza­niumyleth­yl)carbamate monohydrate

**DOI:** 10.1107/S1600536811044850

**Published:** 2011-11-05

**Authors:** Bo Shao, Hai-Bin Wang

**Affiliations:** a College of Biology and Environmental Engineering, Zhejiang Shuren University, Hangzhou 310015, People’s Republic of China; b College of Chemical Engineering and Materials Science, Zhejiang University of Technology, Hangzhou 310014, People’s Republic of China

## Abstract

In the crystal structure of the title compound, C_3_H_8_N_2_O_2_·H_2_O, the organic mol­ecule exists as zwitterion with the carboxyl group deprotonated and the amino group protonated. In the crystal, the components are linked by O—H⋯O and N—H⋯O hydrogen bonds.

## Related literature

CO_2_ readily reacts with amines to yied carbamates, see: Brown & Gray (1982 ▶); Dell’Amico *et al.* (2003) ▶; Jing *et al.* (2007 ▶). For *N*-(2-ammonio­eth­yl)carbamate (AECM), a reactive product of ethyl­enediamine with CO_2_, see: Garbauskas *et al.* (1983 ▶); Antsyshkina *et al.* (2007 ▶). For standard bond lengths, see: Allen *et al.* (1987 ▶). 
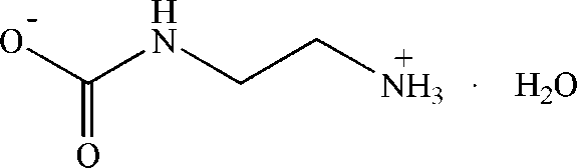


## Experimental

### 

#### Crystal data

C_3_H_8_N_2_O_2_·H_2_O
*M* *_r_* = 122.13
Monoclinic, 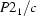

*a* = 8.0301 (6) Å
*b* = 8.7842 (7) Å
*c* = 8.1748 (6) Å
β = 98.889 (1)°
*V* = 569.71 (7) Å^3^
*Z* = 4
Mo *K*α radiation
μ = 0.12 mm^−1^
*T* = 293 K
0.35 × 0.34 × 0.30 mm

#### Data collection

Bruker APEX area-detector diffractometer
Absorption correction: multi-scan (*SADABS*; Bruker, 2001 ▶) *T*_min_ = 0.945, *T*_max_ = 0.966
2877 measured reflections
1002 independent reflections
960 reflections with *I* > 2σ(*I*)
*R*_int_ = 0.016

#### Refinement

*R*[*F* ^2^ > 2σ(*F* ^2^)] = 0.034
*wR*(*F* ^2^) = 0.093
*S* = 1.04
1002 reflections
82 parameters
H atoms treated by a mixture of independent and constrained refinement
Δρ_max_ = 0.21 e Å^−3^
Δρ_min_ = −0.27 e Å^−3^

### 

Data collection: *SMART* (Bruker, 2007 ▶); cell refinement: *SAINT* (Bruker, 2007 ▶); data reduction: *SAINT*; program(s) used to solve structure: *SHELXS97* (Sheldrick, 2008 ▶); program(s) used to refine structure: *SHELXL97* (Sheldrick, 2008 ▶); molecular graphics: *SHELXTL* (Sheldrick, 2008 ▶); software used to prepare material for publication: *SHELXL97*.

## Supplementary Material

Crystal structure: contains datablock(s) global, I. DOI: 10.1107/S1600536811044850/nc2244sup1.cif

Structure factors: contains datablock(s) I. DOI: 10.1107/S1600536811044850/nc2244Isup2.hkl

Supplementary material file. DOI: 10.1107/S1600536811044850/nc2244Isup3.cml

Additional supplementary materials:  crystallographic information; 3D view; checkCIF report

## Figures and Tables

**Table 1:** Hydrogen-bond geometry (Å, °)

*D*—H⋯*A*	*D*—H	H⋯*A*	*D*⋯*A*	*D*—H⋯*A*
O3—H3*A*⋯O1	0.80 (3)	1.92 (3)	2.708 (2)	170 (3)
O3—H3*B*⋯O2^i^	0.86 (3)	1.92 (3)	2.773 (2)	171 (3)
N1—H1*C*⋯O3^ii^	0.89	1.89	2.767 (2)	167
N1—H1*D*⋯O2^iii^	0.89	1.91	2.775 (2)	163
N1—H1*E*⋯O1^iv^	0.89	1.95	2.798 (2)	158
N2—H2⋯O2^v^	0.86	2.43	3.278 (2)	167
C2—H2*A*⋯O1^vi^	0.97	2.56	3.499 (2)	163

Symmetry codes: (i) 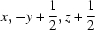; (ii) 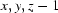; (iii) 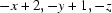; (iv) 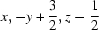; (v) 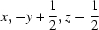; (vi) 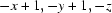.

**Fig. 1.**
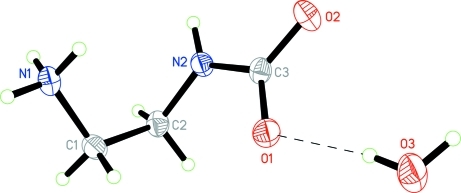
Crystal structure of (I) with labeling and displacemant ellipsoids drawn at the 40% probability level. Intermolecular hydrogen bonding is shown as a dashed line.

**Fig. 2.**
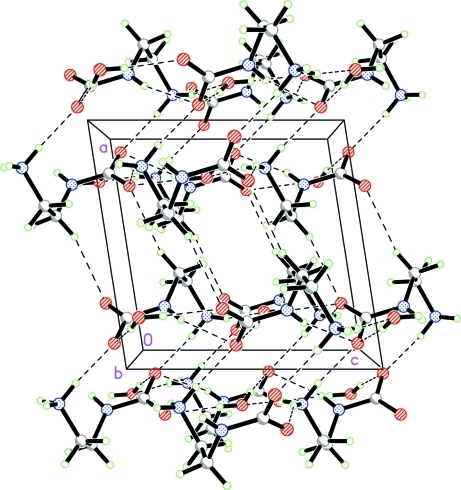
The crystal packing of (I) viewed down the b axis. Hydrogen bonds are drawn as dashed lines.

## supplementary crystallographic information

### Comment

It has been known for decades that CO_2_ readily reacts with amines to yied carbamates (Brown & Gray 1982; Dell'Amico *et al.* 2003; Jing *et al.* 2007). *N*-(2-ammonioethyl)carbamate (AECM), a reactive product of ethylenediamine with CO_2_, was reported previously (Garbauskas *et al.* 1983; Antsyshkina *et al.* 2007). Recently, AECM hydrate, (I) (Scheme 1, Table 1), is prepared from ethylenediamine as starting material in our lab, and its structure is studied hereafter.

In (I), AECM molecule exists as zwitterion, the molecule is linked with the water molecule by an O3—H3A^···^O1 hydrogen bond (Fig. 1, Table 2). The N1 atom is protonated, showing as the center of positive charge. The negative charge is concentrated on the O2 atom of the COO^_^ fragment and is somewhat delocalized: the C3—O1 and C3—N2 bonds are slightly elongated, and the N2—C2 bond is shortened compared to standard values of 1.21, 1.334 and 1.454 Å, respectively (Allen *et al.* 1987). The torsion angle of N1—C1—C2—N2 [46.21 (18)%] is much smaller than that observed in the one of Garbauskas' polymorphs (175.6%), and is smaller than those observed in the second polymorph (66.6% in Antsyshkina's case, 65.5% in Garbauskas' case).

There are many hydrogen bonds in the crystal (Fig. 1, Table 2), playing important role in restraining the AECM comformation, and in building the crystal.

### Experimental

Ethylenediamine (10.1 ml) was dissolved in xylenol (25.2 ml), forming clear solution with stirring, afterwards, the resulting solution was exposed in the air for two month at room temperature. With the reaction deepened, the system separated into two layers gradually. Upper layer was yellowish and pasty, and lower layer was colorless and clear. Crystals of (I) (6.9 g) were at the bottom of the lower lay. Analysis: Cald. for (I) (%): C 29.50, H 8.25, N 22.94; found: C 29.45, H 8.31, N 22.90. IR Spectrum (KBr, cm^-^1): 3289(*s*), 2964(*m*), 2214(w), 1673(*m*), 1601(*s*), 1492(*s*), 1381(*s*), 1332(*s*), 1210(w), 1146(*m*), 1050(w), 1029(w), 1010(w), 887(w), 861(w), 821(*m*), 725(*m*), 646(w), 555(*m*). ^1^H NMR (500 MHz, D2O) δ/p.p.m.: 3.20 (t, 2 H, J = 5.95), 2.97 (t, 2H, J = 5.95).

### Refinement

H atoms of water melecule were deduced from Fourier Maps, and incoporated in refinement freely. The others were placed in calculated positions and allowed to ride on their parent atoms at distances of 0.86Å for acidamide N—H, 0.89Å for amonnium N—H and 0.97Å for ethylene C—H, respectively, with isotropic displacement parameters 1.2–1.5 times *U*_eq_ of the parent atoms.

### Crystal data

**Table:**

C_3_H_8_N_2_O_2_·H_2_O	*F*(000) = 264.0
*M**_r_* = 122.13	*D*_x_ = 1.424 Mg m^−^^3^
Monoclinic, *P*2_1_/*c*	Melting point: 358 K
Hall symbol: -P 2ybc	Mo *K*α radiation, λ = 0.71073 Å
*a* = 8.0301 (6) Å	Cell parameters from 1358 reflections
*b* = 8.7842 (7) Å	θ = 2.4–18.3°
*c* = 8.1748 (6) Å	µ = 0.12 mm^−^^1^
β = 98.889 (1)°	*T* = 293 K
*V* = 569.71 (7) Å^3^	Block, colorless
*Z* = 4	0.35 × 0.34 × 0.30 mm

### Data collection

**Table:**

Bruker APEX area-detector diffractometer	1002 independent reflections
Radiation source: fine-focus sealed tube	960 reflections with *I* > 2σ(*I*)
graphite	*R*_int_ = 0.016
φ and ω scan	θ_max_ = 25.0°, θ_min_ = 2.6°
Absorption correction: multi-scan (*SADABS*; Bruker, 2001)	*h* = −9→9
*T*_min_ = 0.945, *T*_max_ = 0.966	*k* = −10→10
2877 measured reflections	*l* = −9→6

### Refinement

**Table:**

Refinement on *F*^2^	Primary atom site location: structure-invariant direct methods
Least-squares matrix: full	Secondary atom site location: difference Fourier map
*R*[*F*^2^ > 2σ(*F*^2^)] = 0.034	Hydrogen site location: inferred from neighbouring sites
*wR*(*F*^2^) = 0.093	H atoms treated by a mixture of independent and constrained refinement
*S* = 1.04	*w* = 1/[σ^2^(*F*_o_^2^) + (0.0545*P*)^2^ + 0.1863*P*] where *P* = (*F*_o_^2^ + 2*F*_c_^2^)/3
1002 reflections	(Δ/σ)_max_ < 0.001
82 parameters	Δρ_max_ = 0.21 e Å^−^^3^
0 restraints	Δρ_min_ = −0.27 e Å^−^^3^

### Special details

**Table:**

Geometry. All e.s.d.'s (except the e.s.d. in the dihedral angle between two l.s. planes) are estimated using the full covariance matrix. The cell e.s.d.'s are taken into account individually in the estimation of e.s.d.'s in distances, angles and torsion angles; correlations between e.s.d.'s in cell parameters are only used when they are defined by crystal symmetry. An approximate (isotropic) treatment of cell e.s.d.'s is used for estimating e.s.d.'s involving l.s. planes.
Refinement. Refinement of *F*^2^ against ALL reflections. The weighted *R*-factor *wR* and goodness of fit *S* are based on *F*^2^, conventional *R*-factors *R* are based on *F*, with *F* set to zero for negative *F*^2^. The threshold expression of *F*^2^ > σ(*F*^2^) is used only for calculating *R*-factors(gt) *etc*. and is not relevant to the choice of reflections for refinement. *R*-factors based on *F*^2^ are statistically about twice as large as those based on *F*, and *R*- factors based on ALL data will be even larger.

### Fractional atomic coordinates and isotropic or equivalent isotropic displacement parameters (Å^2^)

**Table:**

	*x*	*y*	*z*	*U*_iso_*/*U*_eq_	
O3	0.79493 (17)	0.47441 (13)	0.42240 (14)	0.0480 (4)	
H3A	0.788 (2)	0.472 (2)	0.326 (3)	0.050 (5)*	
H3B	0.844 (3)	0.391 (3)	0.466 (3)	0.069 (6)*	
O2	0.91742 (12)	0.29983 (10)	0.06003 (11)	0.0324 (3)	
O1	0.75733 (13)	0.50450 (10)	0.08899 (11)	0.0325 (3)	
N1	0.82463 (13)	0.69023 (12)	−0.32825 (13)	0.0270 (3)	
H1D	0.9110	0.6736	−0.2480	0.041*	
H1E	0.8288	0.7855	−0.3644	0.041*	
H1C	0.8303	0.6260	−0.4114	0.041*	
N2	0.76650 (14)	0.40847 (12)	−0.16430 (13)	0.0265 (3)	
H2	0.8216	0.3547	−0.2257	0.032*	
C3	0.81591 (15)	0.40494 (13)	0.00256 (15)	0.0235 (3)	
C2	0.62537 (16)	0.49851 (15)	−0.24347 (16)	0.0280 (3)	
H2A	0.5340	0.4898	−0.1790	0.034*	
H2B	0.5862	0.4560	−0.3521	0.034*	
C1	0.66425 (16)	0.66607 (15)	−0.26316 (16)	0.0288 (3)	
H1A	0.5729	0.7126	−0.3380	0.035*	
H1B	0.6709	0.7161	−0.1566	0.035*	

### Atomic displacement parameters (Å^2^)

**Table:**

	*U*^11^	*U*^22^	*U*^33^	*U*^12^	*U*^13^	*U*^23^
O3	0.0844 (9)	0.0333 (6)	0.0250 (6)	0.0148 (6)	0.0046 (6)	−0.0027 (5)
O2	0.0397 (5)	0.0250 (5)	0.0294 (5)	0.0052 (4)	−0.0050 (4)	0.0000 (4)
O1	0.0459 (6)	0.0274 (5)	0.0252 (5)	0.0038 (4)	0.0084 (4)	−0.0035 (4)
N1	0.0343 (6)	0.0222 (5)	0.0239 (5)	−0.0015 (4)	0.0022 (4)	0.0029 (4)
N2	0.0349 (6)	0.0236 (6)	0.0213 (6)	0.0056 (4)	0.0050 (4)	0.0001 (4)
C3	0.0279 (6)	0.0183 (6)	0.0243 (6)	−0.0041 (5)	0.0044 (5)	0.0003 (5)
C2	0.0273 (6)	0.0297 (7)	0.0263 (7)	−0.0017 (5)	0.0018 (5)	0.0038 (5)
C1	0.0316 (7)	0.0263 (7)	0.0283 (7)	0.0062 (5)	0.0036 (5)	0.0023 (5)

### Geometric parameters (Å, °)

**Table:**

O3—H3A	0.78 (2)	N2—C3	1.3608 (16)
O3—H3B	0.88 (3)	N2—C2	1.4499 (16)
O2—C3	1.2725 (15)	N2—H2	0.8600
O1—C3	1.2603 (15)	C2—C1	1.5184 (18)
N1—C1	1.4828 (16)	C2—H2A	0.9700
N1—H1D	0.8900	C2—H2B	0.9700
N1—H1E	0.8900	C1—H1A	0.9700
N1—H1C	0.8900	C1—H1B	0.9700
			
H3A—O3—H3B	110 (2)	N2—C2—C1	114.63 (10)
C1—N1—H1D	109.5	N2—C2—H2A	108.6
C1—N1—H1E	109.5	C1—C2—H2A	108.6
H1D—N1—H1E	109.5	N2—C2—H2B	108.6
C1—N1—H1C	109.5	C1—C2—H2B	108.6
H1D—N1—H1C	109.5	H2A—C2—H2B	107.6
H1E—N1—H1C	109.5	N1—C1—C2	112.39 (10)
C3—N2—C2	123.13 (10)	N1—C1—H1A	109.1
C3—N2—H2	118.4	C2—C1—H1A	109.1
C2—N2—H2	118.4	N1—C1—H1B	109.1
O1—C3—O2	124.74 (11)	C2—C1—H1B	109.1
O1—C3—N2	118.03 (11)	H1A—C1—H1B	107.9
O2—C3—N2	117.23 (11)		
			
C2—N2—C3—O1	−13.44 (17)	C3—N2—C2—C1	79.98 (15)
C2—N2—C3—O2	165.99 (11)	N2—C2—C1—N1	46.09 (15)

### Hydrogen-bond geometry (Å, °)

**Table:**

*D*—H···*A*	*D*—H	H···*A*	*D*···*A*	*D*—H···*A*
O3—H3A···O1	0.80 (3)	1.92 (3)	2.708 (2)	170 (3)
O3—H3B···O2^i^	0.86 (3)	1.92 (3)	2.773 (2)	171 (3)
N1—H1C···O3^ii^	0.89	1.89	2.767 (2)	167.
N1—H1D···O2^iii^	0.89	1.91	2.775 (2)	163.
N1—H1E···O1^iv^	0.89	1.95	2.798 (2)	158.
N2—H2···O2^v^	0.86	2.43	3.278 (2)	167.
C2—H2A···O1^vi^	0.97	2.56	3.499 (2)	163.

Symmetry codes: (i) *x*, −*y*+1/2, *z*+1/2; (ii) *x*, *y*, *z*−1; (iii) −*x*+2, −*y*+1, −*z*; (iv) *x*, −*y*+3/2, *z*−1/2; (v) *x*, −*y*+1/2, *z*−1/2; (vi) −*x*+1, −*y*+1, −*z*.
